# Gastrointestinal complications after cardiac surgery: a retrospective case–control study and risk score development

**DOI:** 10.3389/fcvm.2026.1765841

**Published:** 2026-04-15

**Authors:** Ilaria Giambuzzi, Giorgia Bonalumi, Pietro Messi, Giulia Ballan, Agnese Maccarana, Arianna Galotta, Alice Bonomi, Moreno Naliato, Marco Agrifoglio

**Affiliations:** 1 Department of Cardiac Surgery, Policlinico di Monza, Monza, Italy; 2 Department of Cardiac Surgery, IRCCS Centro Cardiologico Monzino, Milano, Italy; 3 DISCCO Department, University of Milan, Milan, Italy; 4 Unit of Biostatistics, IRCCS Centro Cardiologico Monzino, Milan, Italy; 5 Department of Biomedical, Surgical, and Dental Sciences, University of Milan, Milan, Italy

**Keywords:** cardiac surgery, cardiopulmonary bypass, coronary surgery, gastrointestinal complications, valve surgery

## Abstract

**Introduction:**

Gastrointestinal (GI) complications after cardiac surgery, although uncommon (0.4–3%), are associated with high mortality rates (13–63%). This study aimed to describes outcomes of GI complications and propose a risk score predicting their occurrence.

**Methods:**

We conducted a retrospective case–control study including 8,544 patients undergoing cardiac surgery between 2005 and 2019. GI complications—defined as bleeding, ischemia/perforation, obstruction, or pancreatitis—were used to select cases for 1:2 propensity score matching with controls. Multivariable logistic regression was performed to determine independent predictors of a composite endpoint (postoperative pulmonary, renal, and cardiac complications). A GI complication risk score was then developed.

**Results:**

After matching, 162 patients were analyzed (54 GI group, 108 controls). Smokers and patients with significant coronary artery disease were more frequent in the GI group (*p* = 0.0049 and *p* = 0.0013). GI complications included ischemia (50.6%), hemorrhage (31.5%), pancreatitis (3.7%), and obstruction (14.8%), with 24.1% occurring as part of multiorgan failure. Overall mortality in the GI group was 38.8% compared with 0.9% in controls (*p* < 0.0001). Independent predictors of the composite endpoint were GI complications, NYHA class >2, and preoperative acute myocardial ischemia. The risk score, incorporating smoking, age, chronic kidney disease, and mitral valve replacement, showed good discrimination (area under the curve 0.735, 95% CI 0.653–0.816) and calibration (Hosmer–Lemeshow *p* = 0.934).

**Conclusions:**

Mortality remains high among patients who develop GI complications after cardiac surgery, regardless of treatment modality. This risk score represents a potentially valuable tool for identifying patients at increased risk and underscores the importance of close monitoring and timely intervention in this population.

## Introduction

Gastrointestinal (GI) complications following cardiac surgery with cardiopulmonary bypass (CPB) are uncommon but clinically significant due to their high morbidity and mortality rates (13%–63%). Recent epidemiological data estimate an incidence of 0.5%–4.5% in adult cardiac surgery interventions requiring CPB (1, 2). The severity of these complications is largely attributed to diagnostic challenges and the wide spectrum of clinical presentations. Early postoperative sedation, analgesia, and mechanical ventilation may mask symptoms and signs, contributing to delayed diagnosis and treatment (3). Visceral hypoperfusion—particularly in high-risk patients who suffered from low-output cardiac syndrome and/or hypovolemia, combined with high doses of vasopressor—is currently considered the leading cause of postoperative GI complications (4–8).

Despite their rarity, the difficulty of diagnosis contrasts sharply with the urgent need for early recognition. Timely identification is essential for rapid diagnostic evaluation, prompt therapeutic intervention, and prevention of mortality (4, 9, 10). GI complications encompass a heterogeneous range of entities, from mild conditions amenable to conservative management to severe, late-diagnosed forms associated with life-threatening deterioration.

The most common manifestations include bowel ischemia and gastrointestinal bleeding—most frequently originating from the upper GI tract. Other relevant complications include peptic ulcer disease, intestinal obstruction, pancreatitis, acute cholecystitis, diverticulitis, hyperbilirubinemia, and liver failure (11). Management must be initiated as promptly as possible. Therapeutic strategies vary according to the specific complication and the patient's clinical condition, and may include supportive medical therapy, endoscopic intervention, interventional radiology procedures, or surgery (12, 13). Given the importance of timely diagnosis, greater emphasis should be placed on preoperative variables to facilitate Heart Team discussions and identify patients at increased risk for GI complications. Although several independent predictors of GI complications have been reported in the literature—including concomitant procedures associated with prolonged cross-clamp time, preoperative hypertension, renal failure, immunocompromised status, and a history of heart failure (14)—no comprehensive risk score has yet been developed to support clinicians in the preoperative assessment.

Therefore, the aim of this study was to report the incidence, diagnostic pathways, management strategies, and outcomes of GI complications at our center. Moreover, through a case–control design, we sought to identify independent preoperative predictors of GI complications and develop a risk score to estimate the overall likelihood of their occurrence.

## Methods

We conducted a retrospective study of patients who underwent cardiac surgery between 2005 and 2019. Gastrointestinal complications were defined as bleeding from the GI tract, ischemia/perforation, occlusion, and pancreatitis. The study was approved by the local ethics committee (CCM 1325, 15 July 2020). The data underlying this article are available on reasonable request to the corresponding author. Informed written consent was waived from participants due to the retrospective nature of study. Eligibility included age ≥ 18 years and CPB institution. Exclusion criteria were age < 18 years and emergent or urgent surgery.

Gastrointestinal pathologies were defined as previous GI surgeries, previous GI hemorrhages, diverticulosis or diverticulitis, or previous tumors. Endoscopic procedures included gastroscopy/colonoscopy, while surgical procedures included both laparotomy and laparoscopy. The primary objective of the study was to identify potential independent predictors of GI complications.

### Statistical analysis

Continuous variables were expressed as mean and standard deviation (SD) if normally distributed, or as median and interquartile range (IQR) otherwise. Categorical variables were reported as frequencies and percentages. Continuous variables were compared using Student's *T*-test for independent samples or the Mann–Whitney *U*-test, according to the distribution. The chi-square test or Fisher's exact test was performed to compare categorical data, as appropriate.

A 1:2 propensity score (PS) matching was applied. The PS was calculated using a multivariable logistic regression model using the following variables: age, sex, ejection fraction, coronary artery bypass/no coronary artery bypass, and extracardiac vasculopathy. Matching was performed without replacement, and postmatching balance was assessed using standardized mean differences (SMD) for all covariates. Diabetes was more prevalent in the control group, likely reflecting residual confounding; therefore, it was excluded from variable selection for the risk score.

We constructed a combined endpoint considering the following postoperative complications: pulmonary complications, acute kidney failure, acute myocardial ischemia, neurological complications, and arrhythmias.

Independent predictors of the combined endpoint were assessed by multivariable logistic regression with the stepwise selection method, using a significance level of 0.05 to allow a variable to both enter and stay in the model. We assessed potential collinearity through the variance inflation factor (VIF) among covariates in the multivariable model to confirm the robustness of the model estimates.

We performed a subanalysis in the GI groups using logistic regression to analyze the association between outcome and treatment of GI complications.

A risk score for GI complications was then developed; a logistic regression model was used in which the independent predictors of outcome were selected through cross-validated stepwise logistic regression (excluding diabetes). The risk score was calculated for each subject using the estimated and cross-validated logistic coefficients; the sample was randomly split in half 500 times, and the coefficients were estimated in the first arm (training set), and area under the curve (AUC), sensitivity, and specificity were subsequently tested in the second half (testing set). The mean value of each parameter was considered for the final value. The score was validated by dividing the sample into risk deciles and comparing observed events with predicted events in each decile (Hosmer–Lemeshow test). A calibration plot was generated, and the Brier score was also calculated. The predictive ability of the score was quantified by the area under the ROC curve.

All tests were two-sided, and a *p*-value < 0.05 was considered statistically significant. Statistical analyses were performed using SAS software, version 9.4 (SAS Institute, Cary, NC, USA).

## Results

During the study period, a total of 8,544 patients underwent cardiac surgery at our center. Of these, 54 patients (0.63%) developed a GI complication. After matching, the pool of patients was 162. A total of 54 patients had postoperative GI complications (GI group) and 108 did not have GI complications (Control group). Preoperative characteristics are listed in Table 1. Smokers were significantly more commonly found in the GI group (*p* = 0.0049) as patients with significant coronaropathy (*p* = 0.0013). Nevertheless, no differences were found in previous GI pathology (*p* = 0.8983).

**Table 1:** Preoperative characteristics of patients.

Variable	Control group (108)	GI group (54)	*P*-value	SMD
Age	68.37 ± 7.8	71.3 ± 7.7	0.0250	0.378
Male sex	71 (65.7%)	32 (59.3%)	0.4190	0.134
NYHA > 2	30 (27.8%)	13 (24.1%)	0.6148	0.085
Arterial hypertension	86 (79.6%)	47 (87%)	0.2463	0.200
Smoke	8 (7.4%)	12 (22.2%)	0.0049	0.528
Sinus rhythm	93 (86.1%)	38 (70.4%)	0.0097	0.456
Previous GI pathology	27 (25%)	14 (25.9%)	0.8983	0.021
Vasculopathy	42 (38.9%)	21 (38.9%)	0.9990	<.001
Diabetes mellitus	37 (34.3%)	6 (11.1%)	0.0095	0.589
Previous AMI	25 (23.2%)	12 (22.2%)	0.8947	0.022
Significant coronaropathy	60 (55.5%)	37 (68.5%)	0.1126	0.270
COPD	11 (10.2%)	14 (25.9%)	0.0089	0.418
CKD	7 (6.5%)	9 (16.7%)	0.0405	0.322
LVEF	56.99 ± 11	54.94 ± 11.6	0.2743	0.181

AMI, acute myocardial ischemia; COPD, chronic obstructive pulmonary disease; CKD, chronic kidney disease; LVEF, left ventricular ejection fraction.

Biological mitral valve replacement, biological aortic valve replacement, and ascending aorta replacement were more commonly performed in the GI group (Table 2).

**Table 2:** Surgery most commonly performed in the GI group.

Variable	Control group	GI group	*P*-value	SMD
Biological MVR	2 (1.9%)	11 (20.4%)	0.0001	0.617
Biological AVR	30 (27.8%)	25 (46.3%)	0.0190	0.391
Ascending aorta replacement	3 (2.8%)	7 (13%)	0.0166	0.385

MVR, mitral valve replacement; AVR, aortic valve replacement.

Gastrointestinal complications included ischemia in 27 (50.6%), hemorrhage in 17 (31.48%), pancreatitis in 2 (3.7%), and occlusions in 8 (14.81%) patients. In 13 (24.07%) patients, GI complications occurred in the context of multiorgan failure (MOF). Overall, there were 21 (38.9%) deaths, 12 of whom (57.14%) died as a consequence of MOF. The highest number of deaths was seen in the group with the ischemic complications.

In 15 patients (27.78%), GI surgery was performed and, among them, five patients (33.3%) died as a consequence of an ischemic event. Endoscopic treatment was performed in two patients (3.7%), who both died. Lastly, conservative treatment was performed in 32 patients (59.26%) with 14 deaths (43.75%). The most common cause of death with GI complication as the primary event (nine patients, 42.86%) was ischemia: Four patients (44.44%) were treated conservatively because of diagnostic delay. Two other patients of ischemia were surgically treated and survived. One ischemic patient (small ischemic area) was conservatively treated and was discharged from the hospital on postoperative day 51.

Overall mortality was 34.9% (22 deaths). Mortality in the GI group and control group was 38.8% and 0.9% (*p* < .0001), respectively.

Among the tested variable, GI complications, NYHA >2, and preoperative acute myocardial ischemia were independent predictors of the combined endpoint (Table 3). Collinearity diagnostics (VIF) did not show significant collinearity.

**Table 3:** Independent predictors of postoperative complications.

Variable	OR	95% CI	95% CI
GI complications	6.976	3.005	16.192
NYHA>2	3.149	1.363	7.277
Preoperative AMI	1.988	1.053	3.753

GI, gastrointestinal; AMI, acute myocardial infarction.

Within the GI group, the type of treatment was not associated with mortality (Table 4) or postoperative complications (Table 5). As GI complications were independent predictors of the combined endpoint, we built a score to predict them.

**Table 4:** Odds ratio of treatment of GI complications in relation to mortality.

Variable	OR	95% CI	95% CI
Surgery*	0.909	0.241	3.423
Endoscopy*	0.655	0.108	3.973

*Reference: conservative treatment.

**Table 5:** Odds ratio of treatment for GI complications in relation to morbidity.

Variable	OR	95% CI	95% CI
Surgery*	0.204	0.032	1.278
Endoscopy*	0.444	0.034	5.740

*Reference: conservative treatment.

### Risk score

Independent predictors selected through cross-validation were smoking habit, older age, chronic kidney disease, and mitral valve replacement. These variables were used to create the risk score for GI complications (Table 6), with categorization (0/1/2) defined in Table 7.

**Table 6:** Cross-validated *β* coefficients for the construction of risk score for GI complications.

Variable	*β*
Intercept	−1.939
Mitral valve replacement	5.411
Chronic kidney disease	1.341
Smoking habit	0.964
Age > 70	1.019

**Table 7:** Risk score categorical variables.

Age
0 = ≤ 70 years
1 = > 70 years
Smoking habit
0 = No
1 = Ex-smoker
2 = Active smoker
Chronic kidney disease
0 = No
1 = Yes
Mitral valve replacement
0 = No
1 = Yes

We used the following formula,

$$\mathrm{Score}=\frac{e^{a}}{1+e^{a}}$$

where

a=−1.934+5.411*SVM+1.019*Age+1.341*CKD+0.964*Smoke

For example, a patient younger than 70 years, not smoker, without chronic kidney disease, and without mitral valve replacement scored 0.126, corresponding to an estimated 12.6% probability of developing a GI complication:

a=−1.934+5.411*0(noSVM)+1.019*0(Age≤70)+1.341*0(noCKD)+0.964*0(nosmoke)=−1.934

So,

$$\mathrm{Score}=\frac{e^{-1.934}}{1+e^{-1.934}}=0.126$$

**Figure 1:**
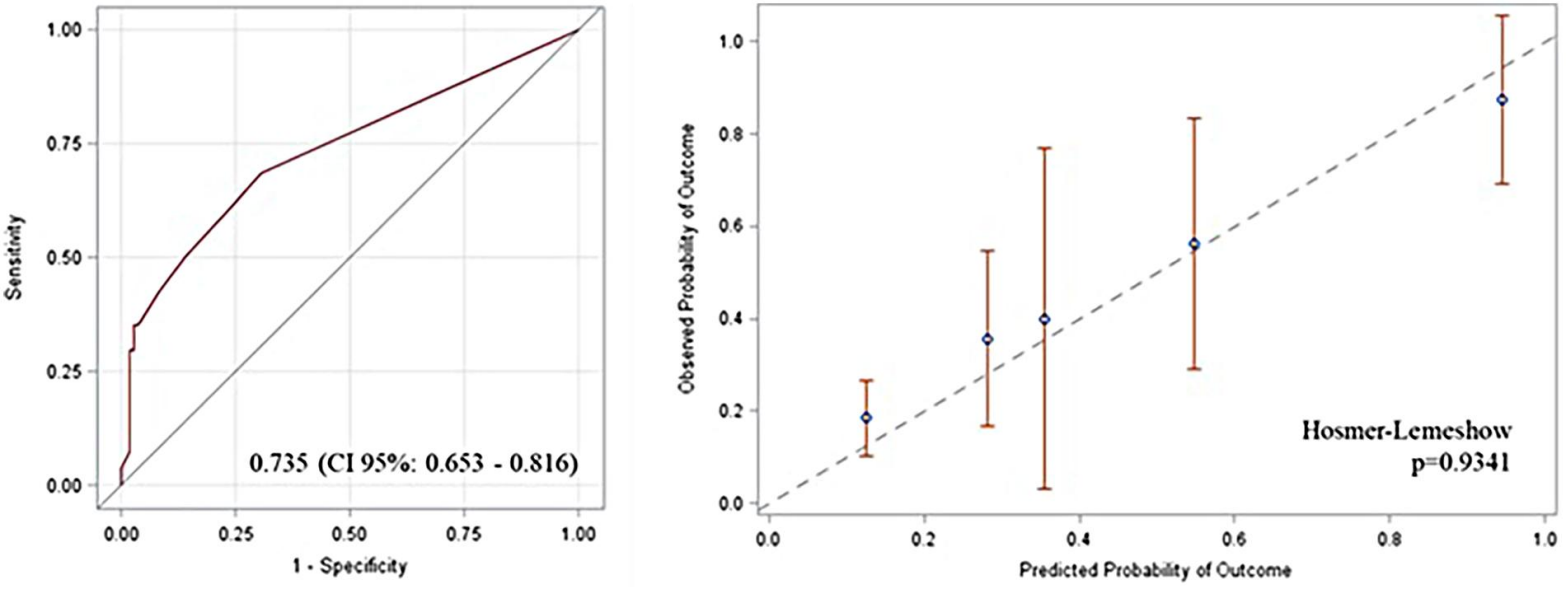
Area under the curve (AUC) and calibration plot in the overall population showing good prediction and good calibration.

Score validation revealed an AUC of 0.735 (CI 95% 0.653–0.816). The calibration plot showed a good calibration (Hosmer–Lemeshow *p* = 0.9341) (Figure 1), with a Brier score of 0.174.

The risk score applied to the GI complications group and the control group yielded a score of 0.13 (0.13–0.27) and 0.32 (0.13–0.74), respectively, with a *p*-value < .0001.

## Discussion

In this retrospective case–control study of 8,544 patients undergoing cardiac surgery with cardiopulmonary bypass, we found that gastrointestinal complications, although infrequent (0.63%), were associated with markedly increased mortality and adverse postoperative outcomes, and we developed a preoperative risk score with moderate discriminatory ability to identify patients at higher risk.

Intra-abdominal complications occur in only a small proportion of patients undergoing cardiac surgery with CPB, yet they are associated with substantial morbidity, increased healthcare costs, and markedly elevated mortality. In our patient population, the overall incidence of GI complications was 0.63%, at the lower end of rates reported in contemporary literature. For example, Naar et al. (14) showed a 0.8% incidence among 6,862 patients in a single-center retrospective study, Yadav et al. (15) observed a 2.2% incidence among 4,417 patients, and Hess et al. (1) reported a 2.4% incidence over 10,285 patients from the STS Registry.

Gastrointestinal complications are therefore rare events, often reflecting a common pathophysiological process (visceral hypoperfusion) (16). They encompass GI bleeding, peptic or duodenal perforation, bowel ischemia, pancreatitis, hepatic dysfunction, acute cholecystitis, and paralytic ileus (17–19).

Visceral hypoperfusion during surgery is considered the predominant mechanism, and ischemic injury to the bowel represents one of the most severe and life-threatening complications after CPB (19–21). Inadequate perfusion may fail to meet metabolic demands, promoting GI complications. Close postoperative monitoring, early diagnosis, and timely intervention are crucial to improve outcomes (22–24).

In our study, overall mortality in the GI group was 34.9% (22 deaths), while mortality in the control group was 0.9% (*p* < .0001). This wide gap, reflecting the clinical burden that GI complications have on the postoperative outcome, is consistent with recent literature. A recent meta-analysis by Duman et al. (25), including 116,105 patients, reported an in-hospital mortality of 25.8% among patient with GI complications after cardiac surgery—over 11 times higher than in patients without GI complications. In our experience, early intervention is critical in the management of GI complications, as 41 patients (74.93%) in our series experienced GI complications before overt MOF. The appearance of any abdominal sign—such as distention, tenderness, or pain—should prompt immediate evaluation by the general surgery team, without waiting for signs of overt perforation and MOF. Appropriate diagnostic timing and close collaboration between cardiac and general surgeons are crucial for identifying complications promptly and enabling earlier, more effective treatment. Suspicion of an abdominal complication should lead directly to early therapeutic intervention, as both endoscopy and abdominal exploration are generally well tolerated in these patients. The increased focus on early diagnosis has contributed to improved outcomes over time (26).

Our patient series highlights several considerations regarding GI complications. First of all, the presence of GI complications, NYHA class >2, and preoperative myocardial ischemia was associated with an increased risk of postoperative adverse events. This finding underscores the well-recognized tendency of GI complications to occur alongside other postoperative complications, reflecting a pattern of MOF. Indeed, Gross et al. (27) reported that patients who had mechanical circulatory support, prolonged mechanical ventilation, and renal replacement therapy were more likely to suffer from GI complications, confirming that often GI complications arise in the context of MOF. Whether GI complications are a consequence of MOF or an initiating factor, however, remains unclear.

Second, although type of surgery was not a significant predictor in logistic regression—likely due to limited sample size—mitral valve replacement was more frequent in the GI complication group and was selected during the stepwise procedure for score development. This finding aligns with previous evidence reporting a higher incidence of GI complications after mitral valve replacement (28). One possible explanation is that inferior vena cava cannulation during this procedure may promote splanchnic venous stasis, increasing the risk of GI complications.

Moreover, the type of intervention (medical, endoscopic, or surgical) likely influences patient outcomes. We did not observe a significant correlation in our analysis, presumably because the therapeutic strategies were appropriately tailored to clinical severity. Conservative treatment was more frequently used in patients with more severe presentations, whereas endoscopic and surgical interventions effectively resolved the complications and prevented mortality.

In the literature, several additional risk factors for GI complications after cardiac surgery have been identified. Groesdonk et al. (29) reported preoperative predictors with high odds ratios, including age >70 years, preoperative renal failure, and diuretic therapy, as well as postoperative factors such as intra-aortic balloon pump use and lactate levels >5 mmol/L. Other studies (30, 31) have identified further predictors, including norepinephrine infusion >0.1 μg/kg/min, ≥ 3 vasoactive medications, prolonged mechanical ventilation, preoperative hemodialysis, hypothermic CPB, advanced age, and preoperative mechanical circulatory support (27). Nevertheless, most of the predictors are intraoperative or postoperative. Our study focuses on preoperative variables, as did that of Naar et al. (14). Indeed, Naar et al. reported hypertension (OR 5.74) and dialysis-dependent renal failure (OR 3.62) as major independent predictors of ischemic gastrointestinal complications. Our findings likewise emphasize baseline clinical vulnerability, identifying chronic kidney disease, age, and smoking as contributors to systemic fragility.

We propose a novel scoring system to assist clinicians in the early identification of patients at risk for GI complications using only preoperative variables. The score demonstrated an area under the ROC curve of 0.735 and incorporates a relatively small number of preoperative variables. Given the low number of events, it was not possible to propose a cutoff-based classification. Therefore, the presence of any of those variables should prompt a more careful assessment of the patient, particularly when any combination is present, as it increases the risk of GI complications. The application of this score may guide clinicians in deciding whether to pursue a more proactive diagnostic evaluation in patients suspected of having GI complications.

## Limitations

Our study has several limitations. To begin with, its retrospective nature limited the availability of information—for example, noradrenaline dosage, lactate levels, or prolonged mechanical ventilation for all patients—which prevented inclusion of these variables in the analysis, unlike previous studies. Furthermore, the small number of patients could introduce a bias into the statistical analysis.

Gastrointestinal complications are fortunately rare, making it difficult to include more patients in the analysis.

We acknowledge that the absence of an observed association between treatment modality (medical, endoscopic, or surgical) and mortality may have been influenced by confounding by indication, as more severe or hemodynamically unstable patients were more likely to receive conservative management. We also recognize that the long study period (2005–2019) may have encompassed significant changes in perioperative management, representing a potential source of heterogeneity that could have affected the results.

Finally, further studies with independent cohorts for external validation are required before clinical application of the risk score. Given the limited number of events and the potential risk of overfitting in the current study, these additional validations are crucial to confirm the reliability and generalizability of the risk score prior to routine use in clinical practice.

## Future perspective

To overcome the methodological limitations of retrospective studies on GI complications—such as small sample size and outdated cohorts—well-designed multicenter prospective studies are warranted. Such studies should integrate detailed hemodynamic parameters with biochemical markers, including data collected during continuous monitoring during CPB.

Emerging evidence also suggests that unmeasured anions may play a significant role in the development of metabolic acidosis (32); therefore, these parameters should be considered when investigating visceral hypoperfusion and GI complications.

Finally, future research should prioritize external validation of the identified predictors and the definition of quantitative cutoffs to enhance preoperative stratification of patients at increased likelihood of GI complications.

## Conclusions

Gastrointestinal complications after cardiac surgery remain an uncommon but clinically relevant condition, frequently occurring in the context of MOF and associated with high mortality. Their impact is amplified by the difficulty of early diagnosis, as clinical signs are often non-specific and diagnostic investigations may be limited by postoperative hemodynamic instability.

The proposed risk score represents a preliminary attempt to stratify the risk of gastrointestinal complications using readily available clinical variables. However, given the retrospective design, the limited number of events, and the lack of external validation, this score should be considered exploratory and hypothesis-generating rather than ready for routine clinical implementation. Nevertheless, the presence of any of the studied risk factors confers a high risk of GI complications. Further prospective studies in larger, independent cohorts are necessary to validate its predictive performance and to clarify its potential role in supporting clinical surveillance and decision-making in high-risk patients.

## Data Availability

The datasets presented in this study can be found in online repositories. The names of the repository/repositories and accession number(s) can be found below: https://zenodo.org/records/17232585.
